# Social capital in association with health status of women in reproductive age: study protocol for a sequential explanatory mixed methods study

**DOI:** 10.1186/1742-4755-11-35

**Published:** 2014-05-09

**Authors:** Azam Baheiraei, Fatemeh Bakouei, Eesa Mohammadi, Mostafa Hosseini

**Affiliations:** 1 Community Based Participatory Research Center, Iranian Institute for Reduction of High-Risk Behaviors, Tehran University of Medical Sciences, Tehran, Iran; 2 Department of Reproductive Health, School of Nursing and Midwifery, Tehran University of Medical Sciences, Tehran, Iran; 3 Department of Nursing, Tarbiat Modares University, Tehran, Iran; 4 Department of Epidemiology and Biostatistics, School of Public Health, Tehran University of Medical Sciences, Tehran, Iran

**Keywords:** Social capital, Women’s health status, Reproductive age, Study protocol, Mixed methods study

## Abstract

**Background:**

Women’s health is a general health priority. Preserving and improving women’s health is not only a basic human right, but it is also essential for the health of all nations. Women’s health in Reproductive age affects long-term health of theirs, their family members, and community. Origins of health inequalities are very complicated. Health outcomes are influenced by biological, social and political factors, so to improve women’s health it is necessary to recognize all these factors. Social capital is one of the social determinants of health that might play a considerable role in health inequalities. The association between social capital and health varies according on the sample studied, the type of health outcome and the context in which it is studied. This mixed methods study was designed to determine and explore of relationship between social capital and health status of women of reproductive age in Tehran (capital city of Iran) with its specific social-cultural characteristics.

**Methods/design:**

This study is sequential explanatory mixed methods study, follow-up explanations variant, with two strands (phases). This design will be implemented in two distinct phases. The first phase is a population-based cross-sectional survey on 770 women of reproductive age residing in any of the 22 municipal districts across Tehran. Based on a need to further understand the quantitative results, researchers will implement a second qualitative phase that is designed to help explain the initial quantitative results. Finally, the researchers will present an interpretation about explanation of quantitative results using the qualitative data.

**Discussion:**

This study promotes women’s health by determining the priorities and designing evidence-based interventions founded on the basic and insightful information provided on social capital and the status of the health of women.

## Background

Women play a key role in the formation, maintenance, and promotion of the health of the family and society. Women’s health is the foundation of the society’s health [1]. Preserving and improving women’s health is not only a basic human right, but it is also essential for the health of all nations [2].

The health of women in countries with low to medium income is complicated by their demographics. Although women live longer than men, they tend to experience poorer health [3]; therefore, national policies and plans should generally address health issues for women by focusing on their effective determinants [4]. James et al. stated that since health is influenced by biological, social and political factors, they should all be taken onto consideration when policies are developed to improve women’s health [5]. Detection of the factors associated with women’s health is important for creation of strategies to decrease the incidence of some diseases, enhance quality of life, and reduce the mortality rate [6].

Inequality in health matters is complicated and can be associated with genetic and biological variables, and also the social structure including income, education level, and occupation. Recent studies have examined the relationship between social capital and health status [7]. Some evidences indicate that the social environment affects the health of the populace. Most of these researchers focused on social capital [8] which is a social determinant of health that contributes to inequality in health issues [9,10].

Social capital is typically defined as a combination of social participation patterns and the social cohesion created by participation [11]. Participation and cohesion are the structure and cognitive components of social capital, respectively [12]. Social capital influences health by assuming mechanisms for more convenient access to information by members of society, promotion of decision-making related to the health, and effects on social norms [13]. It also increases use of health services, provides better access to them, and offers psychological support [14,15].

People living in communities with rich social capital experience more positive social, economic and health outcomes. The association between social capital and health, however, is not always consistent; it varies according to the sample studied, the type of health outcome [16-19], and the context in which it is studied [20]. Contradictory information has prevented researchers from reaching a conclusive result. These contradictions can be attributed to the conceptualization and measuring instrument of social capital [21].

The largest percentage of females in the population of Iran (about 60%) is women of reproductive age; approximately 16.2% of these women live in Tehran [22]. Women’s health issues during this stage of life affect the long-term health of the women, their family members and especially their children [23]. It is necessary to study the status of women’s health to determine existing realities that effect ongoing health programs, including selection of program priorities, determining its objectives, strategies, and implementing of the program [24].

Quantitative researches without qualitative researches cannot alone lead to the policy making, planning, and interventions in women’s health and its promotion. On the other hand, the qualitative researches are not efficient enough to be applied in operative programs due to lack of their generalization to all of the society. However, using a mixed methods study to apply advantages both of quantitative researches (high number of samples and generalization) and qualitative researches (listening to the voices of participants and in-depth elaboration) and as well as providing a more complete image of the subject [25]. In other word, the limitations of one method can be offset by the strengths of another method. Hence, this mixed methods study was designed to investigate the following objectives:

1. Determine of women’s health status of reproductive age

2. Determine of women’s social capital status of reproductive age

3. Determine of association between social capital and health status of women of reproductive age

4. Explore women’s perspective of reproductive age about association between social capital and health status

5. Interpret of how qualitative data to help explain the quantitative results.

## Methods/design

This study is sequential explanatory mixed methods study, follow-up explanations variant, with two strands (phases). This design will be implemented in two distinct phases. The first phase involves collecting and analyzing quantitative data. Base on a need to further understanding the quantitative results, researcher implements a second qualitative phase that is designed to help explain the initial quantitative results [25]. Because of the extension and diversity of the assessed variables in this study, it is probable to obtain some results which need further explanation and cannot be described simply by the quantitative data. Some results that might be considered for follow-up are statistically significant results, statistically no significant results, key significant predictors, surprising results, variables that distinguish between groups, outlier or extreme cases or distinguishing demographic characteristics This design is most useful when the researcher wants to assess trends and relationships with quantitative data but also be able to explain the mechanism or reasons behind the resultant trends [25].

Based on researchers’ recognition about which results will be explained, the second phase of this study (i.e., qualitative phase) will be designed to pursue the results of quantitative phase. Finally, the researchers will present an interpretation about explanation of quantitative results using the qualitative data. The study protocol was passed by the Ethics Committee of the Tehran University of Medical Sciences.

The notation for the current study can be written as (QUAN → qual). This notation indicates in which the researchers implement the two strands in a sequence, the quantitative methods will occur first and has a greater weight in addressing the study’s purpose, and the qualitative methods will follow to help explain the quantitative results.

### First phase (strand): quantitative study

The first strand with greater weight and emphasis is a population-based cross-sectional survey on women of reproductive age (15–49 years) residing in any of the 22 municipal districts across Tehran. The sample size in the current study is 770 people, regarding the studies were conducted on social capital in people with P = 50%, d = 4%, and considering the design effect (df = 1.2%). The sampling technique is multi-stage clustering in which the clusters are 5 geographical zones in Tehran (North, South, East, West, and Center). The number of clusters required for each zone will be determined and one neighborhood will be randomly assigned as the main cluster. Next, using a systematic sampling method, the women of reproductive age in the neighborhood households will be interviewed. If a woman declines to take part, the neighboring household will be invited to participate in the study and complete the questionnaire. The women will be asked to provide their phone numbers if they are ready to participate in qualitative phase of the study. The confidentially of their information will be guaranteed.

### Scales and data collection

In this quantitative strand will be used three questionnaires consist:

1. Social Capital Integrated Questionnaire (SC-IQ) which was designed by World Bank in 2000 for measuring the social capital for developing countries. The questionnaire contains 6 dimensions: groups and networks, trust and solidarity, collective action and cooperation, information and communication, social cohesion and inclusion, and empowerment and political action [26]. The validity and reliability of the questionnaire was measured by Nedjat et al. in Iran through forward-backward translation method [27].

2. The SF-36 questionnaire (Short Form Health Survey) is a general scale of health status. This scale was first designed by Ware et al. to evaluate health of the community, determine health policies, and assess the efficiency of the designed treatment. It consists of 8 health-related concepts/domains: physical, role limitations due to physical problems, bodily pain, general health, vitality, social functioning, role limitations due to emotional problems, and mental health [28]. SF-36 questionnaire was translated in Iran by Montazeri et al., using forward-backward translation method, and it has also been culturally adapted and its validity and reliability determined [29].

3. Socio-demographic questionnaire was designed by research team including: age, ethnicity education level (participant; her father, mother and spouse if married), marital status, family size, and occupation status (participant; her father, mother and spouse if married), sufficiency of income for expenses, and crowding index.

### Data analysis

Data will be analyzed using SPSS-16 through descriptive and inferential statistical methods. At first, researchers will describe the status of social capital and health dimensions with frequency and percentage in term Socio-demographic variables. Then, one-way ANOVA test will be used to investigate the association between dimensions of health and social capital and at the end to avoid effects of the confounding factors and to predict effects of independent variables on dependent ones, the stepwise multiple linear regression will be used.

### Second phase (strand): qualitative study

The second strand with lower weight and emphasis is a qualitative study, conventional content analysis. The approach used for sampling in this phase will be purposeful sampling procedure and the participants are selected based on quantitative results. Therefore, regarding which the primary quantitative result need for further explanation and interpretation and which participant offers the best explanation, the researchers will invite some women (participated in initial quantitative data collection) to take part in qualitative phase of the research. After inviting the women for active and voluntary participation in the individual interviews, the time and location of the semi-structured interviews will be determined. The number of participants will be decided by data saturation. The accurate design of questions for secondary qualitative phase and also type of participants in the qualitative research is not possible before completing the quantitative phase and these depend on extraction of the initial quantitative results [25].

The researchers should identify the results that need further information and use these results to guide the design of the research questions, sample selection and data collection questions for qualitative phase. Hence, this design is considered a kind of emergent approaches in mixed methods studies [25].

### Data analysis

Qualitative data will be analyzed using theme development procedure and data management conducted using the software of MAXQDA 10. First, audio files of the interviews will be transcribed. Then, the researchers will examine the content and extract the face and latent themes. Each transcribed word or expression will be considered to be an individual unit of analysis. Further scrutiny of the transcripts and accompanying interpretative notes may contribute to recognition of the initial relationships among the concepts extracted from the expressions. The notes and codes also help formation of themes. As the interviews progress and the relationships among the themes emerge, it will be possible to recognize patterns and main concepts.

### Integration of quantitative and qualitative data

**Figure 1:**
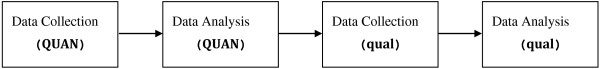
**Visual model of sequential explanatory mixed methods study.** At first, the primary quantitative phase with greater weight and emphasis and then qualitative phase with lower weight and emphasis will perform regarding result of quantitative phase.

**Figure 2:**
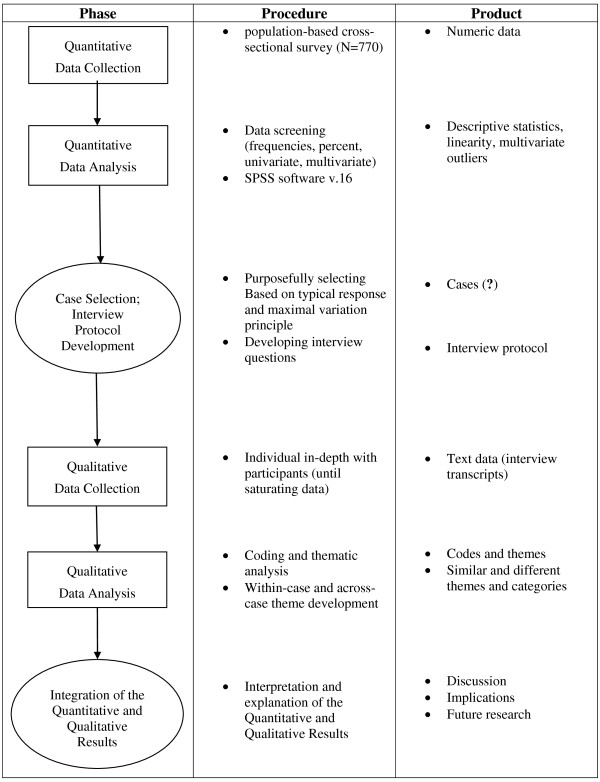
**Diagram for this study that will use the explanatory design.** The quantitative data are collected first and given priority in this study. The qualitative phase will shape based on quantitative results. Therefore, sample selection and interview protocol will develop regarding which the primary quantitative result need for further explanation and interpretation and which participant offers the best explanation. The integration of results occurs in the interpretation and explanation of the quantitative and qualitative results.

At last, researchers will present interpreting of how explain the quantitative results by qualitative data to achieve the objectives of study. Visual model and diagram of this mixed methods study with sequential explanatory design are shown in Figures 1 and 2.

### Ethical issues

Written informed consent will be taken from each participant. The Ethics Committee of the Tehran University of Medical Sciences in Tehran, Iran approved the protocol of this study (code number: 91-04-62-20184).

## Discussion

This study promotes women’s health by determining the priorities and designing evidence-based interventions founded on the basic and insightful information provided on social capital and the status of the health of women. Although previous studies have indicated that the health-associated consequences such as self-reporting [30], cardiovascular diseases [31], mental health [32], and health-related behaviors [8,33,34] are linked with social capital, existing evidence does not unambiguously confirm the useful effects of social capital on social health [17,35]. Further study in this area, particularly in developing countries, is required.

## Abbreviations

QUAN: Quantitative; qual: qualitative; SC-IQ: Social Capital-Integrated Questionnaire; SF-36 questionnaire: Short Form Health Survey-36 questionnaire; SPSS: Statistical Package for Social Science.

## Competing interests

The authors declare that they have no competing interests.

## Authors’ contributions

All the authors contributed to the conception and design of the study. FB drafted the first version of the manuscript. AB, EM, and MH revised the manuscript. AB critically reviewed the manuscript for important intellectual content. All authors approved the final version.

## Authors’ information

Azam Baheiraei: PhD of Health Promotion, Community Based Participatory Research Center, Iranian Institute for Reduction of High-Risk Behaviors, Tehran University of Medical Sciences, Tehran, Iran; Fatemeh Bakouei: PhD Candidate of Reproductive Health, Tehran University of Medical Sciences, Tehran, Iran; Eesa Mohammadi: PhD of Nursing, Department of Nursing, Tarbiat Modares University, Tehran, Iran; Mostafa Hosseini: PhD of Statistics, Department of Epidemiology and Biostatistics, School of Public Health, Tehran University of Medical Sciences, Tehran, Iran.
